# From recognition to response: integrated signaling pathways determining pollen acceptance and rejection in Brassicaceae

**DOI:** 10.1111/nph.70991

**Published:** 2026-02-06

**Authors:** Tong Zhang, Shuyan Li, Shengwei Dou, Qiaohong Duan

**Affiliations:** ^1^ College of Horticulture Science and Engineering Shandong Agricultural University Tai'An 271018 Shandong China; ^2^ Shandong Provincial Key Laboratory of Fruit and Vegetable Germplasm Innovation and Utilization Tai'An 271018 China

**Keywords:** autophagy and ubiquitin‐proteasome pathway, FERONIA receptor, pollen–stigma interaction, RALF–CrRLK1L module, *S*‐locus, stigma barrier, water channel

## Abstract

Generation of competent offspring is vital for the prosperity of flowering plants. The pistil not only functions as a conduit for pollen tubes to grow to the ovary but also provides a selective venue for facilitating the growth of compatible pollen tubes and discouraging invaders and incompatible pollen. This review integrates recent advances in pollen–pistil interactions on dry stigmas of the Brassicaceae in the domains of self‐incompatibility (SI) and cross‐compatibility. We first outline the initial recognition mechanisms that distinguish self from nonself pollen and then highlight how key stigma responses are differentially regulated during compatible and incompatible responses, including calcium signaling, exocytosis, cytoskeleton dynamics, reactive oxygen species, aquaporin activity, and cell wall permeability. By linking these discrete cellular events to their physiological outcomes, we provide a unified framework for understanding how Brassicaceae stigmas precisely control fertilization. A deeper understanding of these mechanisms also informs new strategies for improving crop breeding in economically important Brassicaceae species, which widely use SI to produce F1 hybrid seeds.

**Table:** 

	Contents	
	Summary	788
I.	Introduction	788
II.	The recognition of self‐incompatible pollen	790
III.	Basal compatible responses and their ‘suppression’ in self‐incompatible responses	790
IV.	Stigma barriers actively inhibiting pollen hydration and penetration	793
V.	Conclusions	795
	Acknowledgements	795
	References	796

## I. Introduction

**Fig. 1:**
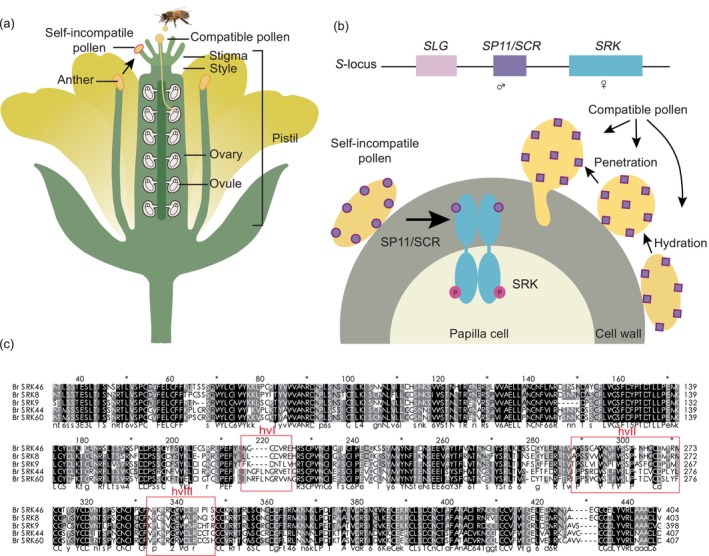
Schematic demonstration of cross‐ and self‐pollination in Brassicaceae. (a) Schematic of a self‐incompatible Brassicaceae flower. Cross‐pollen hydrates, germinates, and penetrates the stigma to complete fertilization, while self‐pollen is rejected at the stigma surface. (b) The *S*‐locus. This comprises the female determinant SRK, the male determinant SP11/SCR, and the modulator *S*‐locus glycoprotein (SLG) (which enhances SRK expression). Haplotype‐specific binding of SP11/SCR to SRK activates the receptor, triggering incompatibility responses to reject self‐pollen. SLG, while not essential for recognition, enhances the self‐incompatibility reaction. (c) Sequence alignment of the extracellular domains of SRKs from different *S*‐haplotypes. The three hypervariable regions (hvI, hvII, and hvIII) critical for specific ligand binding are highlighted in red boxes.

In flowering plants, sexual reproduction is actively regulated by cell–cell interactions between the pollen (male reproductive cells released from the anther) and the pistil (the female reproductive organ; Fig. 1a). Successful fertilization begins at the receptive stigma surface of the pistil, in which pollen hydrates and germinates. The pollen tube then grows extracellularly through the stigma, style, and ovary to deliver sperm cells for double fertilization (Broz & Bedinger, 2021; Zhong *et al*., 2024). Throughout this journey, the pistil functions not merely as a passive conduit for pollen tube growth but also as an active gatekeeper (Bedinger *et al*., 2017; Broz & Bedinger, 2021; Zhong *et al*., 2025). Following pollination, two primary signaling pathways for pollen–pistil recognition are induced in the stigma: the compatibility pathway which ensures acceptance of suitable pollen, and the self‐incompatibility (SI) pathway which prevents inbreeding in outcrossing species (Takayama & Isogai, 2005; Doucet *et al*., 2016; Abhinandan *et al*., 2022). Among the several molecularly characterized SI systems in flowering plants – including gametophytic systems in the Papaveraceae and Solanaceae – the sporophytic system of the Brassicaceae provides a key model for dissecting pollen–stigma interactions at the stigma surface (Goring *et al*., 2023; Bosch & Franklin‐Tong, 2024; Xue, 2025). Notably, even during self‐incompatible pollinations, pollen is still recognized as ‘compatible’ and initiates basal compatible responses in the stigma. However, the self‐incompatible response always dominates, leading to pollen rejection (Jany *et al*., 2019).

Brassicaceae species have dry stigmas lacking abundant exudates; consequently, the hydration of pollen grains can only occur upon release of water and nutrients from the stigma (Dickinson, 1995). In this system, self‐pollen recognition is sporophytically controlled by the multi‐allelic *S*‐locus, which encodes tightly linked male‐ and female determinants (Fig. 1b). These allelic variants, known as *S*‐haplotypes, determine specificity: pollen carrying an *S*‐haplotype identical to that of the stigma, that is pollen from the same or a genetically closely related individual, is recognized as self‐pollen and triggers the SI response (Nasrallah & Nasrallah, 1993; Goring *et al*., 2023). Previous research has established key mechanisms that promote compatible factors during basal compatible responses and their suppression during SI response (Doucet *et al*., 2016; Nasrallah, 2023). Recent findings show that the stigma also constitutively maintains active barriers that inhibit pollen hydration and penetration (Liu *et al*., 2021; Zhang *et al*., 2021; Huang *et al*., 2023; Lan *et al*., 2023). Following pollen–stigma interaction, this barrier is differentially modulated: the recognition of compatible pollen weakens the inhibition to facilitate pollen tube growth, whereas the recognition of self‐incompatible pollen reinforces it (Liu *et al*., 2021; Zhang *et al*., 2021; Lan *et al*., 2023).

The Brassicaceae family includes economically important crops, such as *Brassica rapa*, *Brassica oleracea*, and *Brassica napus*, and various *Arabidopsis* species. While closely related to the outcrossing species *Arabidopsis lyrata*, the model plant *Arabidopsis thaliana* is fully self‐compatible (Nasrallah *et al*., 2004; Vekemans *et al*., 2014; Tsuchimatsu *et al*., 2017). The loss of SI in *A. thaliana* has been attributed to the mutation and deletion of male and/or female determinants (Bechsgaard *et al*., 2006; Shimizu *et al*., 2008; Tsuchimatsu *et al*., 2010, 2017; Vekemans *et al*., 2014). The introduction of functional *S*‐genes from *A. lyrata* or related species into *A. thaliana* has successfully restored its SI system (Nasrallah *et al*., 2002; Boggs *et al*., 2009b; Rea *et al*., 2010; Zhang *et al*., 2019). The transgenic restoration has helped overcome limitations in studying SI in nonmodel *Brassica* species, facilitating research into the molecular and cellular events in stigmatic papillae following compatible and incompatible pollination (Goring *et al*., 2023; Nasrallah, 2023).

In this review, we synthesize the complex molecular and cellular processes of pollen–stigma interactions in the Brassicaceae. As most findings derive from studies in *Brassica* species and *Arabidopsis* species, we refer to genes without specifying species names for conciseness. We focus on three integrated aspects: (1) the initial recognition of self‐incompatible pollen; (2) the regulation of stigma responses that promote compatible factors during basal compatible responses and suppress them during incompatible responses; and (3) the mechanisms by which the stigma barrier actively inhibits pollen hydration and penetration and how this inhibition is dynamically regulated.

## II. The recognition of self‐incompatible pollen

The receptive surface of the stigma is composed of numerous elongated papilla cells, each capable of accepting a pollen grain. Upon landing, proteins and lipids from the pollen coat mix with the stigmatic surface at the contact site, forming a specialized interface known as the ‘pollen foot’. This structure establishes the essential physical and hydraulic connection for pollen adhesion, creating the platform for the subsequent molecular dialogue of recognition (Roberts *et al*., 1980; Dickinson *et al*., 2000).

The recognition of self‐pollen is genetically controlled by a highly polymorphic *S*‐locus encoding tightly linked *S*‐genes expressed in both reproductive tissues (Suzuki *et al*., 1997). SP11/SCR is a small, cysteine‐rich protein localized in the pollen coat (Schopfer *et al*., 1999; Takayama *et al*., 2000). In plants heterozygous for two *S*‐haplotypes with a dominant‐recessive relationship, a pollen grain expresses only the dominant allele due to *trans*‐acting small RNA‐mediated silencing (e.g. Smi and Smi2 in *Brassica*; miR‐based systems in *Arabidopsis*) of recessive SP11/SCR alleles in the diploid tapetum (Tarutani *et al*., 2010; Durand *et al*., 2014; Yasuda *et al*., 2016; Bachmann *et al*., 2021; Dou *et al*., 2021, 2024; Yew *et al*., 2023). SRK (*S*‐locus receptor kinase) is a transmembrane receptor kinase in the papilla plasma membrane that acts as the receptor for its cognate SCR ligand (Stein *et al*., 1991; Goring & Rothstein, 1992; Takasaki *et al*., 2000). Another gene in the *S*‐locus is *S*‐locus glycoprotein (*SLG*), which is expressed in the stigmatic papilla cells. Although its precise role remains under investigation, SLG can enhance the SI response in *B. rapa* (Nasrallah *et al*., 1987; Takayama *et al*., 1987; Kandasamy *et al*., 1989).

At the contact site, this diffusible SCR signal binds with high specificity to its cognate SRK (Kachroo *et al*., 2001; Takayama *et al*., 2001; Shimosato *et al*., 2007). This haplotype‐specific interaction at the pollen–stigma interface is the foundation of the SI response (Fig. 1c). Hypervariable regions (hv) in both SP11/SCR and the extracellular domain of SRK are key for this interaction (Mishima *et al*., 2003; Kemp & Doughty, 2007; Boggs *et al*., 2009a; Ma *et al*., 2016). Structural analysis revealed that SCR interacts simultaneously with hvI and hvII of its cognate SRK, forming a 2 : 2, eSRK : SCR heterotetramer (Ma *et al*., 2016). This complex triggers SRK autophosphorylation and activation, initiating the downstream signaling cascades that lead to pollen rejection (Kachroo *et al*., 2001; Takayama *et al*., 2001). If the pollen is not recognized as self – that is, if the SCR signal and SRK receptor are not from the same *S*‐locus, or if one or both of the *S*‐determinants are mutated or lost – basal compatible responses ultimately promote the hydration and penetration of compatible pollen (Jany *et al*., 2019).

## III. Basal compatible responses and their ‘suppression’ in self‐incompatible responses

### 1. Calcium signaling

Calcium (Ca^2+^) is a fundamental secondary messenger known to regulate pollen tube growth (Lin *et al*., 2025). During pollen–stigma interactions, distinct dynamic changes in stigmatic Ca^2+^ levels are observed (Dearnaley *et al*., 1997; Iwano *et al*., 2004, 2014, 2015), which are thought to be associated with either pollen acceptance or rejection.

**Fig. 2:**
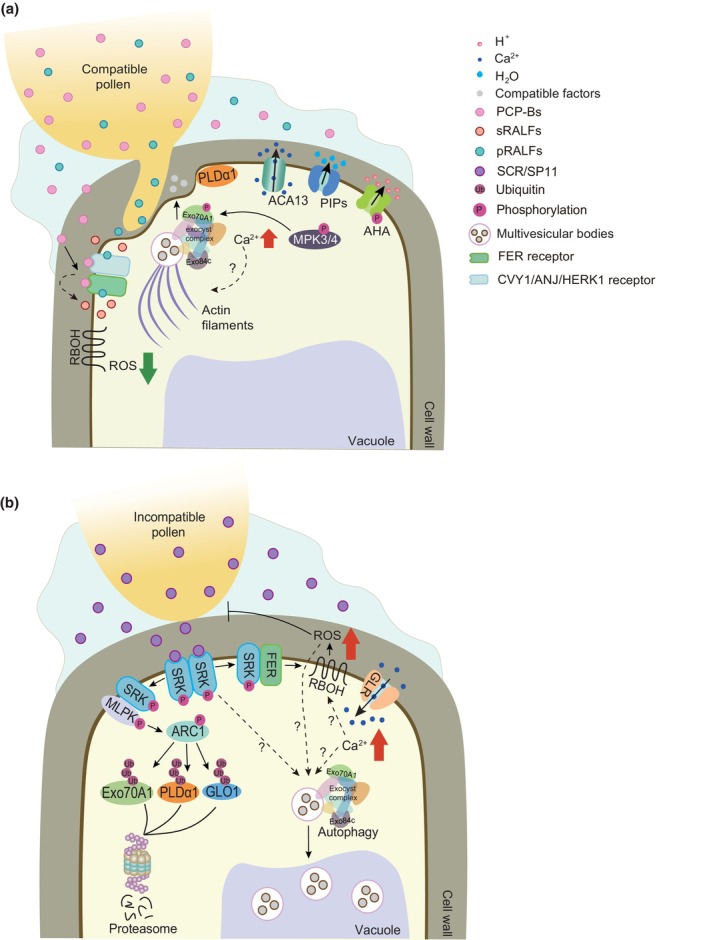
Model of signaling pathways during compatible and self‐incompatible responses in Brassicaceae. (a) Compatible response. A slight increase in papilla cytosolic calcium (Ca^2+^) is observed at the pollen contact site, and ACA13 is supposed to be responsible for Ca^2+^ export to support pollen germination. The compatible factor Exo70A1 is phosphorylated by mitogen‐associated protein kinase 3/4 (MPK3/4) and trafficked to the papilla plasma membrane. PLDα1 produces phosphatidic acid (PA) to facilitate vesicle tethering. Actin filaments are reorganized, a process suggested to support vesicle transport. Pollen‐derived pollen coat‐derived cysteine‐rich peptides (PCP‐Bs) and pRALFs compete for binding to CrRLK1L receptor complexes, leading to a decrease in stigmatic reactive oxygen species (ROS) and promoting pollen tube penetration. Concurrently, plasma membrane H^+^‐ATPases (AHAs) are activated, pumping H^+^ out of the cell. This elevates cytosolic pH, promoting water export through plasma membrane intrinsic proteins (PIPs). (b) Self‐incompatible response. GLR channels mediate Ca^2+^ influx, although the upstream activating signal remains unknown. SCR binding induces SRK autophosphorylation and activation. Activated SRK interacts with *M*‐locus protein kinase (MLPK) at the plasma membrane. Together, they activate the E3 ubiquitin ligase ARM‐repeat containing (ARC1), which targets and degrades downstream compatible components (Exo70A1, PLDα1, and GLO1). Exocyst complex subunits are also degraded via Exo84c‐mediated autophagy. Simultaneously, SRK interacts with FERONIA (FER), which further activates RBOH‐mediated ROS production, blocking pollen hydration and germination. PLD, phospholipase D; GLO1, Glyoxalase 1. Solid arrows represent experimentally confirmed pathways, while dashed arrows represent predicted potential pathways that have not been experimentally verified.

During compatible pollination, given that the Ca^2+^ gradient at the tip region of the pollen tube is essential for its growth, it is speculated that Ca^2+^ is pumped out from the papilla cell (Iwano *et al*., 2014; Lin *et al*., 2025). Experiments coating the *B. rapa* and *A. thaliana* stigmas with the Ca^2+^ indicator Calcium Green revealed elevated Ca^2+^ levels on the papilla surface following cross‐pollination, but not following self‐incompatible pollination. This Ca^2+^ increase is dependent on the Ca^2+^ pump ACA13 (autoinhibited Ca^2+^‐ATPase 13), as mutants lacking ACA13 failed to exhibit this response, leading to impaired pollen germination and reduced seed set. Together, these results indicate that ACA13‐mediated Ca^2+^ export from papillae is essential for compatible pollen growth, although precisely how this surface Ca^2+^ increase facilitates pollen acceptance remains unknown (Iwano *et al*., 2014; Fig. 2a).

During self‐incompatible pollination, *A. thaliana* expressing the Ca^2+^ probe YC3.6 showed a rapid and large Ca^2+^ influx into stigmatic papilla cells, but compatible pollination only triggered a small Ca^2+^ increase (Iwano *et al*., 2015). This influx is mediated primarily by glutamate receptor‐like (GLR) channels (Iwano *et al*., 2015). A *glr3.7* knockout mutant shows a diminished stigmatic Ca^2+^ elevation, while application of GLR agonists (e.g. D‐Ser, D‐Glu) elevated cytosolic [Ca^2+^] levels in wild‐type (WT) *Arabidopsis* (Iwano *et al*., 2015; Fig. 2b). However, how the GLR channels are activated by the initial SCR‐SRK recognition remains unclear.

Thus, Ca^2+^ signaling bifurcates at the stigma: compatible pollination triggers efflux (via ACA13) to nourish the pollen, while incompatible pollination induces influx (via GLRs) to initiate rejection. This Ca^2+^ signal likely acts upstream and integrates with other rejection mechanisms (Bosch & Franklin‐Tong, 2024; Li *et al*., 2024; Zhou *et al*., 2024). Establishing the specific signaling networks in the Brassicaceae during compatibility responses and SI responses remains a key area for future research (Fig. 2b).

### 2. Compatible factors

**Fig. 3:**
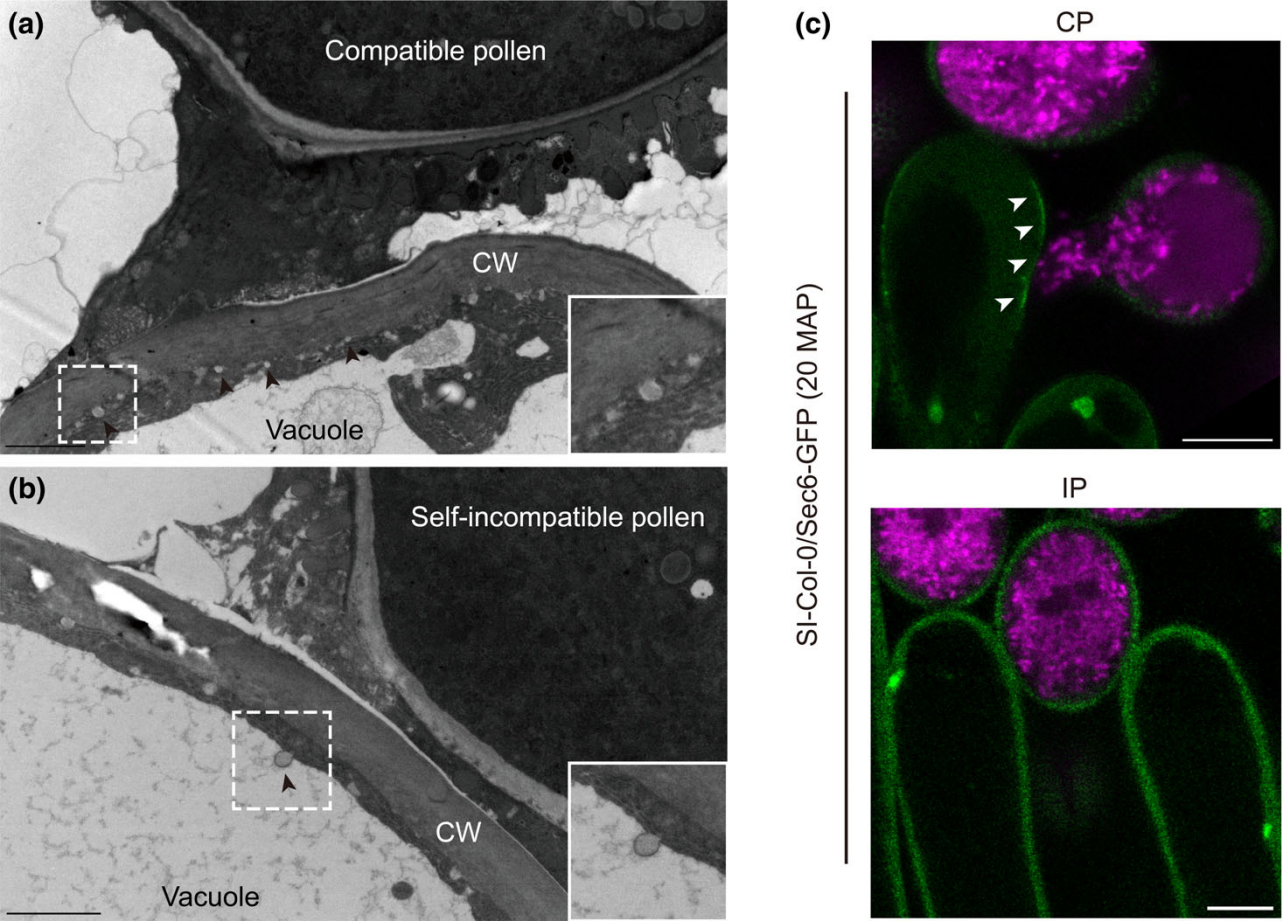
Vesicle trafficking during pollen–stigma interactions in Brassicaceae. (a, b) Transmission electron microscopy (TEM) images of *Brassica napus* stigmatic papillae following compatible (a) and incompatible pollination (b). After compatible pollination, vesicle‐like structures (indicated by arrowheads) are observed fusing to the plasma membrane (PM). Following incompatible pollination, multivesicular bodies or autophagosomes (arrowheads) are observed fusing with the vacuolar membrane, suggesting the degradation of compatible components in the vacuole. The dashed squares indicate the secretory vesicles (a) or multivesicular bodies/autophagosomes (b) are shown in the lower right corner at an enlarged scale. Bars, 2 μm. CW, cell wall. (c) Exocytosis is monitored by Sec6‐GFP upon compatible (CP) and incompatible pollination (IP). The image, reprinted from Zhang *et al*. (2024), shows that exocytosis is activated during compatible pollination, with Sec6‐GFP signal accumulated at the PM adjacent to pollen (white arrowheads). By contrast, the Sec6‐GFP signal is distributed evenly in the cytoplasm after incompatible pollination. Bars, 10 μm. CP, compatible pollination. IP, incompatible pollination. MAP, minutes after pollination. GFP, green fluorescent protein.

Successful hydration and germination of compatible pollen depends on the export of water and compatible factors from papilla cells (Dickinson, 1995). Although the nature of compatible factors remains unknown, they are likely components that promote the compatible responses. It is hypothesized that these compatible factors are delivered to the stigmatic papillae surface by secretory vesicles. Cytoplasmic streaming and vesicle‐like structures have been observed in *B. oleracea* stigmatic papillae upon compatible response (Dickinson, 1995; Elleman & Dickinson, 1996; Fig. 3a,b). However, these secretory events are specifically abrogated upon pollination with self‐incompatible pollen, effectively halting the delivery of resources to the pollen grain (Elleman *et al*., 1992; Safavian & Goring, 2013; Fig. 2a,b). Thus, the regulation of vesicle‐mediated secretion represents a key cellular checkpoint determining pollen fate.

#### Regulation of compatible factors in compatible responses

The secretory vesicles delivering compatible factors rely on the exocyst complex – a complex containing eight subunits, Sec3, Sec5, Sec6, Sec8, Sec10, Sec15, Exo70, and Exo84 – for tethering to the plasma membrane of stigmatic papilla (Chong *et al*., 2010). Live‐cell imaging shows that Sec6‐GFP‐ and GFP‐Exo70A1‐labelled exocytic vesicles accumulate at the papilla plasma membrane adjacent to pollen grains during compatible, but not incompatible, pollinations (Zhang *et al*., 2024; Fig. 3c). The trafficking of Exo70A1 is activated following its phosphorylation by mitogen‐associated protein kinase 3 (MPK3)/4 (Jamshed *et al*., 2020; Fig. 2a). Silencing *Exo70A1* or other subunits of the exocyst complex impairs compatible pollen hydration in *A. thaliana* (Samuel *et al*., 2009; Safavian *et al*., 2015), confirming the function of exocyst complex in vesicle transport during compatible pollen hydration.

Vesicle fusion is further regulated by phospholipase D (PLD), which produces phosphatidic acid (PA), a key messenger for vesicle fusion to target membrane (Ammar *et al*., 2013). PLDα1 activity is crucial for hydration and pollen tube germination in compatible responses (Scandola & Samuel, 2019). Overexpressing PLDα1 or exogenous application of PA to *B. napus* stigmas breaks down the SI and promotes the growth of incompatible pollen tubes into the papillae (Scandola & Samuel, 2019; Fig. 2a). Optimal PLDα1 activity requires cytosolic Ca^2+^, suggesting that a compatible pollination‐induced Ca^2+^ increase may activate PLDα1 to promote vesicle fusion (Hong *et al*., 2016). However, the specific cargos transported by these vesicles and the upstream signals that initiate their delivery remain unidentified.

In addition to vesicle trafficking, papilla cells must maintain a suitable intracellular environment. Glyoxalase 1 (GLO1), which detoxifies the byproduct of glycolysis (methylglyoxal, MG), is identified as another key compatible factor (Sankaranarayanan *et al*., 2015). Overexpression of *GLO1* partially breaks down SI of *B. napus*, while its silencing reduces seed set (Sankaranarayanan *et al*., 2015). It remains unclear whether GLO1 activity is upregulated after compatible pollination and how it is regulated.

#### Degradation of compatible factors in self‐incompatible responses

In contrast to the compatible response, the secretory system responsible for delivering compatible factors is inhibited in stigmatic papillae after incompatible pollination (Elleman *et al*., 1992; Safavian & Goring, 2013; Zhang *et al*., 2024). This inhibition is executed through two parallel degradation pathways targeting compatible factors.

One is the ubiquitin‐proteasome pathway. Following the recognition of self‐incompatible pollen, the activated SP11/SCR‐SRK complex triggers signaling events in the stigmatic papilla that cause pollen rejection (Fig. 2b). The downstream signaling proteins have proven to be rather elusive and so far, only two SRK‐interacting proteins were shown to function in this response: the *M*‐locus protein kinase (MLPK) and the ARM‐repeat containing 1 (ARC1) E3 ligase (Murase *et al*., 2004; Abhinandan *et al*., 2022). MLPK is phosphorylated by SRK after incompatible pollination (Murase *et al*., 2004; Kakita *et al*., 2007; Chen *et al*., 2019). Then, SRK and MLPK together phosphorylate ARC1 (Samuel *et al*., 2009; Scandola & Samuel, 2019). After phosphorylation, activated ARC1 relocates to the proteasome, mediating the ubiquitin‐dependent degradation of specific downstream compatible components, including Exo70A1, PLDα1, and GLO1 (Stone *et al*., 2003; Samuel *et al*., 2008; Abhinandan *et al*., 2022; Fig. 2b). The degradation of GLO1 leads to cytotoxic MG accumulation (Sankaranarayanan *et al*., 2015). Knocking down or knocking out *ARC1* breaks down the SI response in both *B. napus* and *Arabidopsis* (Stone *et al*., 1999; Abhinandan *et al*., 2023), confirming the role of the ubiquitin‐proteasome pathway in pollen rejection during SI.

The other is the autophagy‐mediated vacuolar degradation. Autophagy is a major inducible degradation pathway that participates in plant development and stress response (Zhuang *et al*., 2024). This pathway is also recruited during self‐incompatible pollination, which induces the accumulation of autophagosomes in the vacuole (Safavian & Goring, 2013; Goring, 2017). Loss‐of‐function mutants of the key autophagy genes ATG5 and ATG7 partially break down the self‐incompatible response in *A. thaliana* (Macgregor *et al*., 2022). Recent work identified the exocyst complex subunit Exo84c as a critical mediator of this pathway. Exo84c promotes the autophagic degradation of other exocyst complex subunits (e.g. Sec6, Exo70A1) upon incompatible pollination. An *exo84c* knockout mutant delays the degradation of Sec6 and Exo70A1 (Zhang *et al*., 2024). This demonstrates that the exocyst complex subunits are the direct targets of autophagy during SI responses (Fig. 2b).

While autophagy is clearly engaged, its upstream induction signal in self‐incompatible responses remains unknown. Given that intracellular Ca^2+^ is a key regulator of autophagy in mammals (Høyer‐Hansen *et al*., 2007; Decuypere *et al*., 2011; Li *et al*., 2017; Saikia & Joseph, 2021; Zheng *et al*., 2022; Liu *et al*., 2023), it will be interesting to investigate whether cytosolic Ca^2+^ promotes autophagy in Brassicaceae self‐incompatible responses.

#### Cytoskeleton

The plant cytoskeleton, comprising actin filaments and microtubules, plays central roles in cell shape maintenance, vesicle trafficking, cell division, primary cell wall assembly, and immunity (Chebli *et al*., 2021; Sinha *et al*., 2024; Schoofs *et al*., 2025).

During compatible pollinations, papilla cells rapidly reorganize their actin cytoskeleton. In *B. rapa*, rhodamine‐phalloidin staining reveals that actin bundles polymerize and accumulate in the cytoplasm of papilla cells adjacent to pollen grains (Iwano *et al*., 2007). Similar actin dynamic changes have also been observed in *Arabidopsis* expressing LifeAct‐Venus (Rozier *et al*., 2020; Fig. 2a). This reorganization is absent in papillae cells during incompatible pollination (Iwano *et al*., 2007). Disrupting actin dynamics with cytochalasin D inhibits compatible pollen hydration and germination, confirming that actin polymerization is essential for promoting a compatible response (Iwano *et al*., 2007).

The functional significance of this actin remodeling is likely twofold. First, it may facilitate vesicle trafficking required for exocytosis of compatible factors. In *B. rapa* papillae, compatible pollination induces actin polymerization associated with an apical vacuole network, which is suggested to support pollen hydration (Iwano *et al*., 2007). Second, it could represent a mechanosensitive response to the pressure at the pollen tube penetration site, similar to actin accumulation at pathogen contact sites (Takemoto & Hardham, 2004; Hardham *et al*., 2008).

Microtubules have also been proposed to participate in pollen–stigma interactions. In *Arabidopsis*, the orientation of cortical microtubules regulates the direction of pollen tube growth on stigmatic papillae by influencing the deposition of cellulose microfibrils in the cell wall (Riglet *et al*., 2020). Mutants for KATANIN 1 (*ktn1*), a microtubule‐severing enzyme, exhibit disordered cortical microtubules and cellulose microfibrils. This leads to a loss of mechanical anisotropy in the papilla cell wall, which causes compatible pollen tubes to coil around the papillae instead of penetrating them. A similar coiled phenotype was observed in aged WT papillae with disordered cellulose microfibril pattern, confirming the importance of microtubule‐guided wall mechanics for proper pollen tube guidance (Riglet *et al*., 2020).

This cytoskeletal remodeling is likely coordinated with Ca^2+^ signaling. A well‐established reciprocal crosstalk exists, where the cytoskeleton influences Ca^2+^ homeostasis, and Ca^2+^ signals, in turn, regulate cytoskeletal dynamics (Naveed *et al*., 2024). This interaction could integrate the mechanical and chemical signals during compatibility. The availability of live‐cell markers for both actin/microtubule and secretory vesicles now enables direct testing of this model to determine how cytoskeletal reorganization affects vesicle delivery.

## IV. Stigma barriers actively inhibiting pollen hydration and penetration

Conceptually, beyond the suppression of basal compatible responses during self‐incompatible responses, the stigma also imposes selective barriers actively inhibiting pollen hydration and pollen tube entry. These barriers are maintained by stigmatic signaling modules that are weakened by specific pollen‐derived compatible signals and reinforced by specific pollen‐derived incompatible signals.

### 1. Basal barrier maintenance via endogenous sRALF–CrRLK1L modules

A key regulatory node involves the *Catharanthus roseus* receptor‐like kinase 1‐like (CrRLK1L) members, FERONIA (FER)/SIRÈNE and ANJEA (ANJ), which, together with LORELEI‐like GPI‐anchored protein 1 (LLG1), form receptor complexes on the papilla cell surface (Huck *et al*., 2003; Rotman *et al*., 2003; Escobar‐Restrepo *et al*., 2007; Duan *et al*., 2010, 2014, 2020; Li *et al*., 2015; Cheung, 2024). These complexes bind the stigma autocrine RAPID ALKALINIZATION FACTOR 23/33 (sRALF23/33) peptides and maintain a basal inhibitory stigma barrier before pollination (Liu *et al*., 2021; Zhang *et al*., 2021; Lan *et al*., 2023). The sRALF–CrRLK1L interaction maintains this barrier by initiating three pathways involving reactive oxygen species (ROS) accumulation, aquaporin gating (water channel), and cell wall permeability.

#### Reactive oxygen species accumulation

ROS plays important roles in diverse physiological processes, but high levels of ROS burst are a common defense mechanism against pathogens (Qi *et al*., 2017; Otulak‐Kozieł *et al*., 2020). In the stigma, the sRALF–CrRLK1L interaction activates downstream NADPH oxidase RESPIRATORY‐BURST OXIDASE HOMOLOG D (RBOHD), leading to constitutive accumulation of ROS. This basal level of ROS in unpollinated stigmas is inhibitory for pollen hydration (Liu *et al*., 2021; Zhang *et al*., 2021). Accordingly, stigmas of *fer‐4* mutant or *rbohD* mutant, which have reduced levels of stigmatic ROS, exhibit faster hydration of compatible pollen (Liu *et al*., 2021; Zhang *et al*., 2021).

#### Aquaporin closure

On the dry stigma of Brassicaceae, pollen hydration depends on effective water transport from the stigmatic papilla cells, a process facilitated by plasma membrane aquaporins, such as plasma membrane intrinsic proteins (PIPs; Windari *et al*., 2021). Notably, many PIPs are gated by cytosolic pH, undergoing conformational closure upon protonation (Törnroth‐Horsefield *et al*., 2006; Anderberg *et al*., 2011; Groszmann *et al*., 2017). The sRALF–CrRLK1L module inhibits plasma membrane H^+^‐ATPases (AHAs), preventing proton efflux. The resulting lower cytosolic pH promotes the protonation and closure of aquaporins (e.g. PIP1;2), effectively blocking water release (Liu *et al*., 2025). Consistent with this, *ralf33* mutant stigmas, which have a higher stigmatic pH before pollination, facilitate faster pollen hydration (Liu *et al*., 2025).

#### Cell wall remodeling

After pollen germination, the pollen tube must penetrate the papilla cell wall. FER and other CrRLK1L family members are known to be important for maintaining cell wall integrity (Cheung & Wu, 2011; Duan *et al*., 2020; Liu *et al*., 2024). The cell wall barrier is maintained by a broader CrRLK1L module involving FER, ANJ, HERCULES1 (HERK1), CURVY1 (CVY1), together with four stigmatic autocrine RALF peptides (sRALFs: RALF1, RALF22, RALF23, and RALF33; Lan *et al*., 2023). Stigmas of mutants deficient in key components – such as *fer*, *cvy1*, *anj*, *herk1*, and a *ralf* quad mutant (which carries mutations in all four sRALFs) – are unable to maintain the cell wall barrier, allowing the penetration of interspecific pollen tubes that would be rejected by WT stigmas (Lan *et al*., 2023).

A second layer of control involves the transcription factor STIGMATIC PRIVACY 2 (SPRI2) and its homolog SPRI2‐like, which promote the expression of the cell wall biosynthesis enzymes TBL40 and TBL45. The finding that mutants for either SPRI2 or TBL genes compromise the barrier – allowing interspecific pollen tube penetration – directly implicates this transcriptional module in fortifying the stigma cell wall during pollination (Fujii *et al*., 2023).

### 2. Weakening the barrier by compatible pollen‐derived signals

To allow pollen hydration and penetration, the stigma barriers – ROS accumulation, aquaporin closure and cell wall permeability – must be weakened. This process is triggered by signals from compatible pollen.

#### Reducing ROS

Following compatible pollination, a rapid reduction in stigmatic ROS is essential for successful pollen hydration and pollen tube growth (Liu *et al*., 2021; Zhang *et al*., 2021). Compatible pollen employs at least three strategies to achieve this.

Pollen coat‐derived cysteine‐rich peptides (PCP‐Bs) are crucial regulators of pollen hydration (Wang *et al*., 2017). PCP‐Bs compete with stigma RALF23/33 for binding to CrRLK1L receptors, preventing the activation of RBOHD‐mediated ROS production and thus leading to pollen hydration (Wang *et al*., 2017; Liu *et al*., 2021; Fig. 2a). PCP‐B peptides also trigger high levels of nitric oxide (NO) in the stigma, which is intimately engaged with ROS (Domingos *et al*., 2015). Stigma NO triggered by compatible pollen inactivates FER and RBOHD via nitrosation, shutting down the core ROS‐production machinery (Huang *et al*., 2023). Scavenging stigmatic NO causes ROS accumulation and inhibits compatible pollen tube growth (Huang *et al*., 2023). Furthermore, ROS homeostasis is regulated by several scavenging enzymes. For example, knocking out *Ascorbate peroxidase 1* (*APX1*), a key enzyme that detoxifies hydrogen peroxide (H_2_O_2_), in *B. napus* stigmas leads to delayed compatible pollen hydration (Liang *et al*., 2024). However, how the activity of scavenging enzymes is regulated during compatibility responses remains unclear. The structural mechanisms by which PCP‐Bs outcompete sRALFs for binding with the receptor complex await elucidation.

#### Opening aquaporin water channels

Water channels are also opened by signals from compatible pollen. The replacement of sRALFs by PCP‐Bs activates plasma membrane AHA activity, which pumps protons out of the papilla cell. This elevates the cytosolic pH near the plasma membrane, causing deprotonation and the opening of aquaporins (PIP1/2s). Targeted water efflux is thereby to facilitate pollen hydration (Wang *et al*., 2017; Liu *et al*., 2021; Liu *et al*., 2025; Fig. 2a).

#### Loosening the cell wall barrier

A distinct set of pollen‐derived RALF peptides (pRALFs) targets the cell wall permeability maintained by the sRALFs–CrRLK1L module. These pRALFs compete with sRALFs (RALF1, RALF22, RALF23, RALF33) for binding to a set of CrRLK1L receptors (FER/ANJ/HERK1/CVY1). This ligand switch is proposed to modify the papilla cell wall, possibly through interactions with leucine‐rich repeat extensin (LRX) proteins, making it locally permeable to pollen tube penetration (Lan *et al*., 2023; Zhong *et al*., 2025; Fig. 2a). In practice, compatible pollen or application of pRALFs from compatible pollen helps interspecific pollen overcome the hybridization barrier on the stigma. Future research to elucidate the complete structures of these ligand–receptor complexes will reveal how the RALF–CrRLK1L–LLG module is assembled and how ligand–receptor specificity is achieved.

### 3. Reinforcing the barrier by signals from incompatible pollen

In contrast to compatibility, self‐incompatible or interspecific pollination actively reinforces the barrier. The binding of incompatible pollen signals – such as the self‐pollen ligand SCR or the interspecific pollen signal SRK‐interacting Interspecific Pollen Signal – to the stigma SRK receptor enhances the interaction between SRK and FER. This rapidly activates the ROS‐production pathway, triggering a strong oxidative burst that irreversibly arrests incompatible pollen (Zhang *et al*., 2021; Huang *et al*., 2023; Cao *et al*., 2025; Fig. 2b). In practice, spraying stigmas with ROS scavengers effectively breaks down the self‐incompatible response, permitting the growth of incompatible pollen and enabling seed production from otherwise sterile self‐incompatible inbred lines (Zhang *et al*., 2021).

The inhibitory effect of the elevated stigmatic ROS on pollen was demonstrated by a ‘stigma transfer experiment’ (Zhang *et al*., 2021). In this assay, stigmas were pollinated with self‐pollen to induce ROS elevation and then transferred at different time points (0–60 min) to a culture medium containing ROS scavengers. The results showed a sharp, time‐dependent decline in rescue: immediate transfer (0 min) allowed *c*. 80 self‐pollen tubes to grow, but delays of 15 and 30 min reduced this number to only 42 and 10 tubes, respectively. This stands in contrast to the complete rejection of self‐pollen observed in nontransferred, self‐incompatible stigma (Zhang *et al*., 2021). These results suggest that prolonged exposure to high ROS levels irreversibly arrests self‐pollen development on the stigma.

Mechanistically, the FER‐mediated activation of ROS production may be linked to a broader signaling cascade. In other contexts, stress‐induced increases in cytosolic Ca^2+^ are known to activate NADPH oxidases (Arfaoui *et al*., 2018; Ravi *et al*., 2023). It is therefore plausible that during the SI response in Brassicaceae, Ca^2+^ influx into papilla cells serves as the upstream signal that induces the ROS burst.

An important open question is whether signals from self‐incompatible pollen and interspecific pollen also enhance the water channel and the stigma cell wall barrier. Ca^2+^ influx is suggested to be the upstream signal for cytosolic acidification, which might be responsible for aquaporin inactivation in rejecting undesired pollen (Bosch & Franklin‐Tong, 2024; Wang *et al*., 2024, 2025). Future studies should determine whether these pathways are similarly amplified during incompatibility responses.

## V. Conclusions

In the Brassicaceae, pollen–stigma interactions leading to either acceptance or rejection follow a conserved sequence: Initial signal recognition is followed by the execution of a specific cellular response.

The compatible response orchestrates pollen acceptance by coordinating the stigmatic supply of water and essential factors with the active dismantling of inhibitory barriers, including reducing ROS and weakening the cell wall. Conversely, the incompatible response utilizes a parallel yet opposing pathway, blocking this supportive supply while simultaneously amplifying inhibitory signals to enforce pollen rejection.

Strikingly, the signaling architecture is not unique to the Brassicaceae stigmas but shares deep similarities with the SI system in Papaveraceae and with the plant innate immune system (Kessler *et al*., 2010; Goring *et al*., 2023). Despite utilizing distinct ligand–receptor pairs for initial recognition, these pathways converge on conserved downstream events, including Ca^2+^ influx, ROS bursts, cytoskeletal remodeling, and targeted trafficking (Qi *et al*., 2017; Wang *et al*., 2022; Goring *et al*., 2023; Ravi *et al*., 2023; Bosch & Franklin‐Tong, 2024; Naveed *et al*., 2024). This mechanistic overlap suggests plants that employ a modular and economical signaling toolkit to respond to diverse external stimuli, whether from pollen or pathogens. Elucidating the evolutionary origin of these SI systems, whether potentially derived from ancient defense‐related recognition pathways, will be a compelling challenge for the future.

Despite significant progress, key questions remain. The molecular basis for how SI signaling decisively overrides compatible pathways is still unknown, and the cross‐regulation between the various downstream cellular events is poorly understood. A more comprehensive understanding of these processes is not only fundamental to plant biology but also of direct practical importance. SI is a widely used tool in breeding programs to produce F_1_ hybrid seeds. Elucidating its mechanisms will therefore provide new strategies for manipulating fertility and improving crop breeding in economically important Brassicaceae species.

## Competing interests

None declared.

## Disclaimer

The New Phytologist Foundation remains neutral with regard to jurisdictional claims in maps and in any institutional affiliations.
